# Repurposing of *Bryophyllum pinnatum* for dysmenorrhea treatment: a systematic scoping review and case series

**DOI:** 10.3389/fphar.2023.1292919

**Published:** 2023-12-01

**Authors:** Leonie Zurfluh, Marialuigia Giovannini Spinelli, Cornelia Betschart, Ana Paula Simões-Wüst

**Affiliations:** ^1^ Department of Obstetrics, University Hospital Zurich, University of Zurich, Zurich, Switzerland; ^2^ Klinik Arlesheim, Research Department, Arlesheim, Switzerland; ^3^ Praxis Geburt & Familie, Dr. med. Werner Stadlmayr GmbH, Aarau, Switzerland; ^4^ Department of Gynecology, University Hospital and University Zurich, Zurich, Switzerland

**Keywords:** dysmenorrhea, pain, inflammation, contractility, smooth muscle, *Bryophyllum pinnatum*, *Kalanchoe pinnata*

## Abstract

Dysmenorrhea affects women throughout their reproductive years but there has been a lack of effective and well-tolerated treatment options. Pain symptoms mainly result from inflammatory processes and increased contractile activity in the myometrium. The reported use of *Bryophyllum pinnatum* preparations against inflammation and pain in ethnomedicine as well as current pharmacological data on their inhibition of myometrial contractility led us to hypothesize that this medicinal plant might be a new treatment option for dysmenorrhea. In the first part of the present work, clinical, *in vivo,* and *in vitro* studies on the anti-nociceptive and anti-inflammatory, as well as on myometrium relaxing properties of *B. pinnatum* are reviewed. In the second part, cases of five women with dysmenorrhea who were tentatively treated with a *B. pinnatum* product are described. The review revealed thirty-three experimental *in vivo* and *in vitro* studies, but no clinical study, reporting anti-nociceptive and anti-inflammatory effects of *B. pinnatum* extracts and compounds in a wide range of conditions. Moreover, sixteen publications on smooth muscle contractility revealed relaxing effects. The latter consisted of clinical evidence, as well as of *in vivo* and *in vitro* data. The evidence reviewed therefore provided a rational basis for the use of *B. pinnatum* in the treatment of dysmenorrhea. We subsequently set out to tentatively treat patients with a well-tolerated *B. pinnatum* product that is registered (without indication) and commonly used in obstetrics and gynecology in Switzerland. All five treated patients reported a reduction in pain symptoms and 4 out of 5 indicated a reduced intake of painkillers during menstruation. Taken together, the reviewed information on the pharmacological properties and clinical evidence of *B. pinnatum* extracts and compounds as well as the outcomes of all five patients in the case series support our hypothesis in favor of *B. pinnatum* as a new, well-tolerated therapeutic approach for dysmenorrhea. Prospective clinical studies are urgently needed.

## 1 Introduction

### 1.1 Dysmenorrhea

Dysmenorrhea–derived from the Greek, meaning “difficult menstrual flow” (Deligeoroglou, 2000)–is defined as painful uterine cramps shortly before and/or during menstruation that typically last for 8–27 h. Abdominal pain is usually most severe on the first day of menstruation, may radiate to the back and thighs and may be accompanied by other symptoms like nausea, vomiting, diarrhea and fatigue (Hofmeyr and Bassin, 1996; Ruoff and Lema, 2003). Dysmenorrhea has a major impact on the everyday life of affected women, leading to decreased work productivity and a drop in social activities (Ortiz et al., 2009; Eryilmaz et al., 2010; Wong and Khoo, 2010; Pitangui et al., 2013; Smith and Kaunitz, 2022). The disorder is divided into two subtypes: primary dysmenorrhea, where there is no underlying uterine pathology, and secondary dysmenorrhea with similar pain symptoms but due to disorders like endometriosis, adenomyosis or uterine fibroids (Smith and Kaunitz, 2022). First occurrence of primary dysmenorrhea takes place at or shortly (6–24 months) after menarche and the disorder often affects women throughout their reproductive years (Dawood, 2006; Smith and Kaunitz, 2022).

Risk factors for experiencing primary dysmenorrhea are early menarche, nulliparity, irregular menstrual cycle, prolonged and heavy menstrual bleeding, family history of dysmenorrhea, and smoking. Due to diverse definitions and a lack of assessment tools, data on prevalence ranges from 45% to 95% of menstruating women (Jamieson and Steege, 1996; Proctor and Farquhar, 2006; Unsal et al., 2010) whereby in 5%–15% of cases the symptoms interfere with normal daily activities (Pinkerton, 2020). Part of the prevalence data are thought to be underestimated since healthcare professionals as well as women affected themselves disregard the disorder and think of it as normal part of the menstrual cycle, regardless of the severe distress it causes (Jamieson and Steege, 1996; Proctor and Farquhar, 2006; Unsal et al., 2010).

**FIGURE 1:**
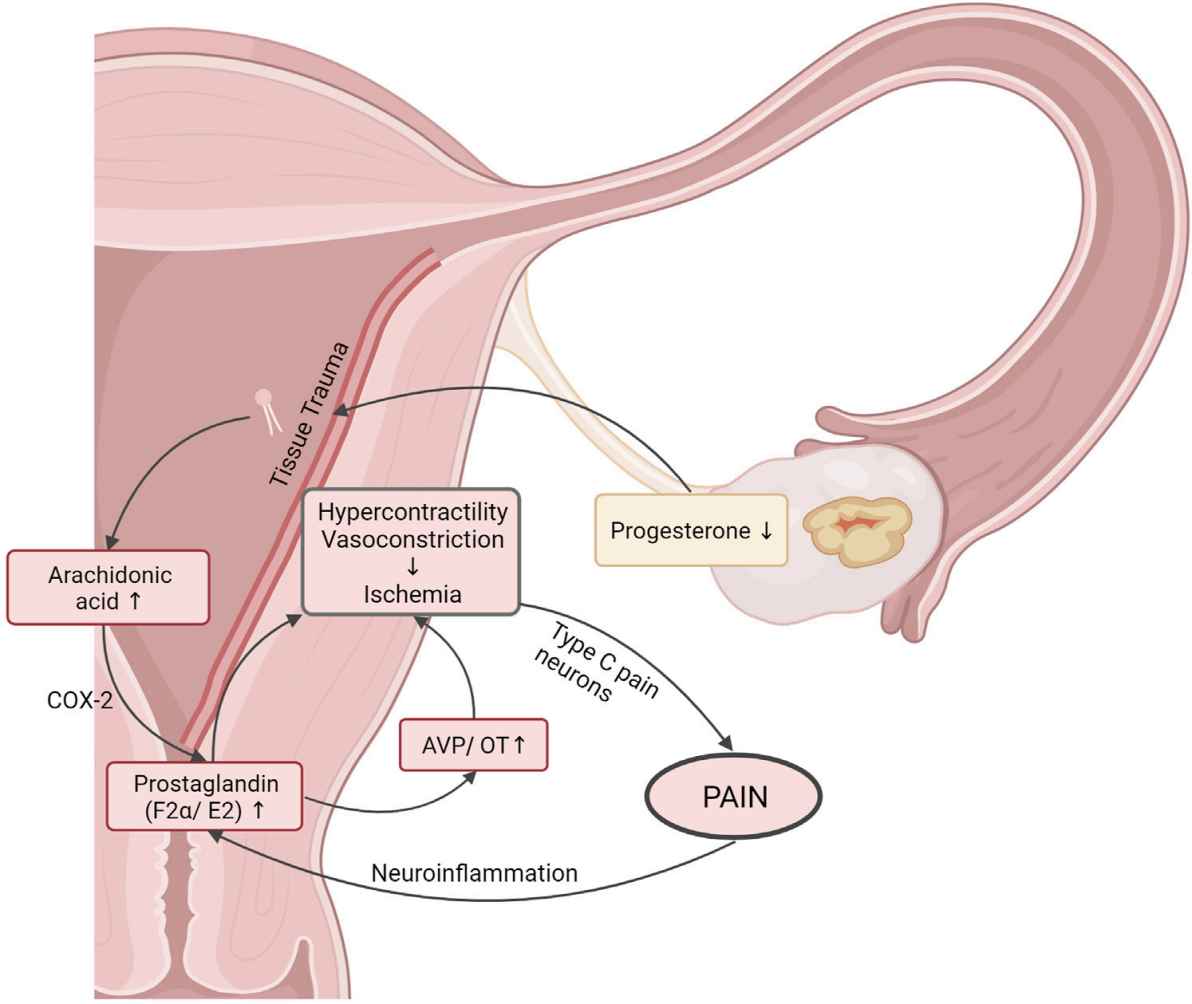
Simplified diagram of the pathophysiology of primary dysmenorrhea. For details, see text; the figure was created with BioRender.com.

The etiology of pain symptoms that women with primary dysmenorrhea experience mainly results from uterine contractions, the increased contractile activity being caused by various biological processes that are depicted in Figure 1 (Iacovides et al., 2015). The non-rhythmic and uncoordinated contractions lead to high uterine pressure, which leads to reduced blood flow. Furthermore, myometrial blood flow is also impaired due to vasoconstriction in the myometrial tissue. Thus, uterine ischemia develops, leading to stimulation of type C pain neurons and to the typical painful sensation of abdominal menstrual pain (Dawood, 2006). The overproduction of prostaglandins (PG), especially F2α and E2, in the dysmenorrheic uterus is believed to play a major role in the pathophysiology of dysmenorrhea. These inflammatory mediators are responsible not only for inflammation, but also for pain, the increase in body temperature, and sleep dysregulation. Physiologically, prostaglandins are produced from arachidonic acid in a reaction catalyzed by the enzyme cyclooxygenase 2 (COX-2) (Iacovides et al., 2015). The arachidonic acid itself is cleaved from phospholipids by phospholipase A. The decrease in progesterone in the late luteal phase of the menstrual cycle leads to an increased release of phospholipids, as does the tissue trauma due to rejection of mucous tissue in the uterus (Iacovides et al., 2015). Although all women have elevated PGF2α levels during menstruation, dysmenorrheic women have even higher levels and the level of PGF2α correlates with pain intensity (Lundström and Green, 1978). Additionally, regular perception of pain bears the risk of chronification of pain symptoms, which is presumed to occur due to structural and functional changes in the brain, and neuroinflammation (Vergne-Salle and Bertin, 2021; Schrepf et al., 2023). These changes are especially important in primary dysmenorrhea as it typically starts at a very young age.

Treatment options for dysmenorrhea include pain medication such as paracetamol or non-steroidal anti-inflammatory drugs (NSAID) or–especially but not only if contraception is desired–hormonal contraceptives. A cross-sectional study among female university students revealed that 70.5% take NSAID to manage pelvic pain during menstruation (Karout et al., 2021). In a review including 80 randomized controlled trials, NSAID were found to be almost 5 times more effective than placebo to relieve abdominal pain and more effective than paracetamol (Marjoribanks et al., 2003). However, NSAID are not a solution for every patient due to the frequent occurrence of NSAID-resistant dysmenorrhea. Up to 18% of women report non-existent or insufficient pain relief, a phenomenon in which underlying mechanisms are still not fully understood (Oladosu et al., 2018). Furthermore, even if painkillers like ibuprofen are effective, they carry the risk of side effects, especially if taken frequently. In the meta-analysis mentioned before, (severe) adverse effects like gastrointestinal problems, headaches and drowsiness underlined the limitation of this treatment option (Marjoribanks et al., 2003). Due to the lack of safe and effective therapy options in conventional medicine, patients with pelvic pain seek help via complementary and alternative therapies like acupressure, heat application (Karout et al., 2021) and herbal medicine (Slavin et al., 2010; Luo et al., 2023). New well-tolerated and effective therapies are urgently needed.

### 1.2 *Bryophyllum pinnatum*

The succulent herb *Bryophyllum pinnatum* (Lam.) Oken. (Crassulaceae) (synonym: *Kalanchoe pinnata* Lam. Pers.) originally only grew in Madagascar, but is at present widely found in tropical and subtropical regions of Africa and Asia. The species is rich in secondary plant metabolites including flavonoids, triterpenes, bufadienolides, steroids and phenanthrenes among others (Hamburger et al., 2017). The main groups of secondary metabolites found in *B. pinnatum* leaves are flavonoid glycosides and bufadienolides (Fürer et al., 2013; Oufir et al., 2015). The presence of nine different glycosides of quercetin, kaempferol, myricetin, acacetin, and diosmetin, and four bufadienolides (bersaldegenin-1-acetate, bryophyllin A, bersaldegenin-3-acetate, and bersaldegenin-1,3,5-orthoacetate) was shown in *B. pinnatum* press juice (Fürer et al., 2013). The most abundant flavonoid in *B. pinnatum* leaves and flowers, namely, quercetin 3-O-α-L-arabinopyranosyl (1→2) α-L-rhamnopyranoside (Coutinho et al., 2012; Fürer et al., 2013), shows a rather unusual and unique glycosylation pattern that is typical to *Bryophyllum* species (Fürer et al., 2013; Dos Santos Nascimento et al., 2018).

The traditional use of *B. pinnatum* and related species in tropical countries goes far back and its range of indications is wide: treatment of wounds, bruises and insect bites (Agoha, 1973); gastrointestinal diseases such as diarrhea, flatulence and vomiting due to its astringent effects (Kamboj and Saluja, 2009); hypertension and urinary disorders (Lans, 2006). Anti-inflammatory, anti-bacterial and anti-viral effects of preparations from *B. pinnatum* seem to support some of these uses (Kolodziejczyk-Czepas and Stochmal, 2017). In the integrative approach of Anthroposophic Medicine, *B. pinnatum* was introduced in the treatment of preterm contractions in the 1970s (Hassauer et al., 1985; Daub, 1988; Fürer et al., 2016). Today, *B. pinnatum* preparations are also used in conventional settings in Switzerland, mainly in gynecology and obstetrics; besides preterm contractions, also overactive bladder syndrome, nocturia and sleeping disorders are common indications (Fürer et al., 2015b; Schenkel et al., 2018; Gantner et al., 2021).

The reported use of *B. pinnatum* preparations against pain and inflammation in ethnomedicine as well as in the treatment of preterm contractions led us to hypothesize that *B. pinnatum* might be a new treatment option for primary dysmenorrhea. In the first part of the present work, clinical and experimental data on anti-inflammatory and anti-nociceptive properties of *B. pinnatum* as well as on its myometrium-relaxing effects, are reviewed according to an *a priori* defined protocol. In the second part, the first five cases of women with dysmenorrhea who were tentatively treated with a well-tolerated *B. pinnatum* product that is registered and commonly used in Switzerland (Bryophyllum 50% chewable tablets by Weleda AG) are described. Finally, the reviewed evidence is discussed in view of dysmenorrhea pathophysiology.

## 2 Methods

### 2.1 Literature review

**FIGURE 2:**
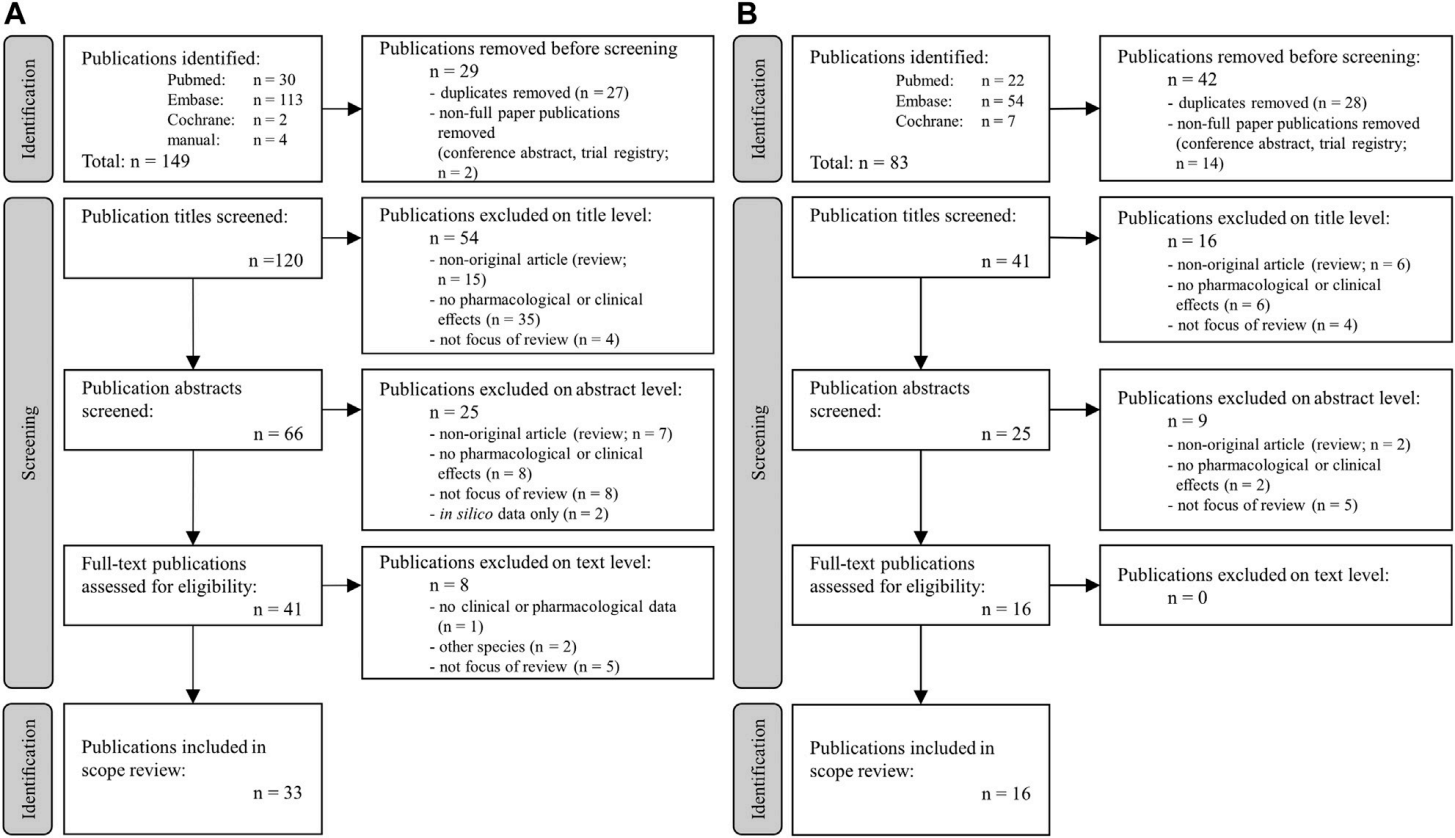
Flow chart of the review selection process on **(A)** anti-nociceptive and anti-inflammatory effects and **(B)** anti-contractility and smooth muscle relaxing effects of *B. pinnatum.*

This scoping review was performed according to the Preferred Reporting Items for Systematic Reviews and Meta-Analyses extension for Scoping Reviews (PRISMA-ScR) guidelines according to the *a priori* protocol described below. The flowchart of the selection process is presented in Figure 2A, B. The research question addressed in the review was: do *B. pinnatum* extracts or compounds have anti-nociceptive/anti-inflammatory and smooth muscle relaxing effects?

#### 2.1.1 Search strategy

The literature review was split into two search strategies: a search of records on anti-inflammatory and anti-nociceptive effects of *B. pinnatum* was performed using the search terms “(“Bryophyllum pinnatum” OR “Kalanchoe pinnata”) AND ((inflamm* OR (cyclooxygenase OR COX) OR prostaglandin OR MAPK OR NFkB) OR (antinocicep* OR pain OR analgesic))”; the search regarding the smooth muscle relaxing effects of *B. pinnatum* was carried out using the search terms “(“Bryophyllum pinnatum” OR “Kalanchoe pinnata”) AND (“smooth muscle” OR myometr* OR bladder OR vessel OR relax* OR contract* OR tocolys*)”. Databases searched were PubMed, Embase and Cochrane. The final search was conducted on 10 July 2023.

#### 2.1.2 Study selection

The study selection was performed independently by LZ and MGS; different classification of the studies was solved by discussion with APSW. Citations were listed and duplicates were removed. Since only full-text articles were taken into consideration, congress abstracts and entries in trial registries were also removed. The remaining records were screened for relevance first on the level of the title, followed by the abstract and then full-text whereas ineligible publications were removed due to the following exclusion criteria: species other than *B. pinnatum*; non-original articles; no quantitative data on pharmacological or clinical outcome measures; *in silico* data only; general anti-oxidative effects only; not focus of scoping review. An Excel list and Endnote 20^®^ were used for data management during the screening process.

#### 2.1.3 Data extraction

Data extraction was first performed by LZ and in a second step complemented/verified by APSW. The following information was extracted for each publication: year of publication, type of study, plant material, pathophysiological focus and major findings. Retrieved data was grouped into clinical, *in vivo* and *in vitro* data.

### 2.2 Case series

In this consecutive case series, five patients with dysmenorrhea were experimentally treated with Bryophyllum 50% chewable tablets; brief case descriptions are presented below. The typical sequence of events was as follows: 1) patient presented at the medical practice with dysmenorrhea complaints; 2) treatment with Bryophyllum 50% chewable tablets was initiated after patients’ informed consent; 3) clinical follow-up, allowing for a comparison of the outcomes before and after the Bryophyllum 50% chewable tablets treatment. Patients were asked to rate their pain using a numeric rating scale (NRS; from 1 = no pain, to 10 = worst pain) twice and to report on the standard pain medication used and absences from work due to dysmenorrhea symptoms.

#### 2.2.1 Medication

Patients were treated with Bryophyllum 50% chewable tablets (à 350 mg, each corresponding to 170 mg of fresh leaf press juice) manufactured by Weleda AG, Arlesheim, Switzerland with *B. pinnatum* leaves provided by Weleda, Brazil. Medication was dosed upon physicians’ choice whereby previous studies on long-term treatments served as orientation points (2-2-2 for overactive bladder (Betschart et al., 2013), and 0-0-2-2 for nocturia (Mirzayeva et al., 2023)). A voucher specimen (No. ZSS 29717) has been deposited at the Zurich Succulent Plant collection, Switzerland. The chewable tablets are registered at the Swiss Agency for Therapeutic Products (Swissmedic; without indication).

#### 2.2.2 Ethics

Written consent for publication of their case was obtained from all five patients. A waiver for specific ethics authorization was given for the study publication of this case series, as it does not fall under the jurisdiction of the Swiss Federal Law on data protection (Human Research Act, Article 2)

## 3 Results

### 3.1 Literature review

#### 3.1.1 Overview of the current literature and of the included articles

Despite a marked relative increase of the literature on *B. pinnatum* during the last decade (34 publications found with the keywords “Bryophyllum AND pinnatum” between 1971 and 2012 and 69 between 2013 and 2023), a manageable number of publications was found. This encouraged us to proceed with the initially planned two searching strategies on the (i) anti-nociceptive and anti-inflammatory and (ii) relaxant/contractility-inhibitory properties of *B. pinnatum* (Figure 2A, B, respectively). Approximately a quarter of the initially found publications were reviews. Since there was no limit regarding publication year, only original publications—no systematic reviews—were included in the scoping review. No case report was identified in the search process. The search strategy restricted the work to publications in English or at least with an English summary. There were distinctly more articles on *in vitro* and *in vivo* data but only a limited amount on clinical studies. Due to the variety of approaches, no classical risk/quality assessment was performed.

Thirty-three experimental studies reporting on anti-nociceptive and anti-inflammatory effects and sixteen publications reporting on relaxing effects on smooth muscle contractility of *B. pinnatum* extracts and compounds were included in the review after the selection process. In the following subsections, the data found from the two searches have been synthesized (see Tables 1, 2). For each search, data have been grouped by topic/indication and paragraphs mentioning clinical evidence first, followed by results from animal models and *in vitro* experimental evidence.

**TABLE 1:** Methodological characteristics, plant material and main results of included publications on anti-nociceptive and anti-inflammatory effects of *B. pinnatum*.

Reference	Title	Type of study	*Bryophyllum pinnatum* material	Main outcome assessment	Main results
Pal and Nag Chaudhuri (1990)	Anti-inflammatory action of *Bryophyllum pinnatum* leaf extract	*In vivo* inflammation rodent models	Methanolic leaf extract	(A) Effect on carrageenan-induced paw edema	Results obtained at 300 mg/kg i.p. (A) 87.8% inhibition of paw edema
				(B) Effect on peritoneal inflammation and capillary permeability induced by acetic acid	(B) inhibitory effect on peritoneal inflammation shown by reduction of total protein in exudate and inhibited capillary leakage
				(C) Effect on cotton pellet granuloma	(C) 38.7% inhibition of granulation tissue development
				(D) Effect in chronic arthritis models	(D) 83.3% reduction of inflammation in arthritis models
Pal and Nag Chaudhuri (1991)	Studies on the anti-ulcer activity of a *Bryophyllum pinnatum* leaf extract in experimental animals	*In vivo* gastric lesion rodent models	Methanolic fraction of leaf extract	(A) Anti-ulcer activity on substance- and stress-induced gastric lesions	Results obtained at 100 and 300 mg/kg i.p. (A) Anti-ulcer activity in nine different experimental animals models
				(B) Ulcer healing effect in acetic acid-induced gastric lesions	(B) Enhancement of the healing process in acetic acid-induced chronic gastric lesions
Pal and Nag Chaudhuri (1992)	Further studies on the anti-inflammatory profile of the methanolic fraction of the fresh leaf extract of *Bryophyllum pinnatum*	*In vivo* inflammation rodent models	Methanolic leaf extract	(A) Effect on carrageenan-induced granuloma	Results obtained at 300 mg/kg (A) inhibition of granuloma development by 67.5% (s.c. application)
				(B) Effect on picryl chloride induced ear edema	(B) Inhibition primary irritation (50% inhibition) and delayed hypersensitivity (i.p. application)
				(C) Effect on arachidonic acid-induced paw edema	(C) Inhibition of paw edema (i.p. application)
				(D) anti-oxidant effect on glucose oxidase-induced inflammation	(D) anti-oxidant effect by inhibition of release of oxygen containing radicals (i.p. application)
Olajide et al. (1998)	Analgesic, anti-inflammatory and antipyretic effects of *Bryophyllum pinnatum*	*In vivo* inflammation and pain rodent models	Methanolic leaf extract	(A) Anti-inflammatory effect in carrageenan-induced paw edema	Results obtained after 50–200 mg/kg i.p. (A) Inhibition of paw edema (52%–66%) (B) Reduction of granuloma development (32% inhibition at 200 mg/kg/d) (C) Dose-dependent temperature reduction (D) Dose-dependent inhibition of writhing (11%–67%)
				(B) Anti-inflammatory effect in cotton-pellet granuloma	
				(C) Antipyretic activity in brewer’s yeast-induced pyrexia	
				(D) Anti-nociceptive activity in acetic-acid induced writhing	
Pal et al. (1999)	Neuropsychopharmacological profile of the methanolic fraction of *Bryophyllum pinnatum* leaf extract	*In vivo* inflammation and pain rodent models	Methanolic leaf extract	(A) Analgesic effect in acetic acid-induced abdominal constriction model	Results obtained at 100 mg/kg i.p. (A) Reduction of writhing (25%)
				(B) Analgesic effect in tail clip method	(B) No analgesic effect in the tail-clip model
Igwe and Akunyili (2005)	Analgesic effects of aqueous extracts of the leaves of *Bryophyllum pinnatum*	*In vivo* inflammation and pain models in mice	Aqueous leaf extract	(A) Definition of LD_50_	Results obtained at 300 mg/kg p.o. (A) No severe toxic effects (LD_50_ = 660.9 mg/kg body weight)
				(B) Effect on pain threshold in hot plate method	(B) Dose-dependent increase of pain threshold by 193.5%
				(C) Effect on pain threshold in phenylbenzoquinone-induced writhing model	(C) Reduction of writhing by 80%
Ojewole (2005)	Antinociceptive, anti-inflammatory and antidiabetic effects of *Bryophyllum pinnatum* (Crassulaceae) leaf aqueous extract	*In vivo* inflammation and pain rodent models	Aqueous leaf extract	(A) Anti-nociceptive effect in hot-plate and acetic acid model	(A) Dose-dependent anti-nociceptive effect at 25–800 mg/kg i.p (B) Inhibition of acute inflammation by 30% (90 min after treatment with 400 mg/kg p.o.)
				(B) Anti-inflammatory effect in paw edema model	(C) Caused hypoglycemia in normoglycemic and diabetic rats at 25–800 mg/kg p.o
				(C) Antidiabetic effect in streptozotocin-induced diabetes mellitus model	
Gupta et al. (2010)	Anti-inflammatory activity of the leaf extracts/fractions of *Bryophyllum pinnatum* Saliv.Syn	*In vivo* inflammation model in rats	Various leaf extracts and fractions	Anti-inflammatory effect of extract and fractions on formaldehyde-induced paw edema model in rats	Inhibition of paw edema; methanolic extract was most effective leading to 64% inhibition at 500 mg/kg
Afzal et al. (2012)	Anti-inflammatory and analgesic potential of a novel steroidal derivative from *Bryophyllum pinnatum*	*In vivo* inflammation and pain rodent models	Aqueous leaf extract and isolated compound (urs stigmast-4, 20 (21), 23-trien-3-one)	(A) Carrageenan-induced paw edema model	(A) Extract led to reduction of paw edema by 87% (400 mg/kg p.o.) compound led to reduction of paw edema by 84% (300 mg/kg p.o.)
				(B) Acetic acid induced writhing model	(B) Extract let to 80% protection against writhing (400 mg/kg i.p.) compound led to 75% protection against writhing (300 mg/kg, i.p.)
Chaturvedi et al. (2012)	Pharmacognostical, phytochemical evaluation and antiinflammatory activity of stem of *Kalanchoe pinnata* Pers	*In vivo* inflammation rodent models	Stem extract not further characterized	(A) Acetic acid-induced vascular permeability	(A) Reduction of permeability (51% at 400 mg p.o.)
				(B) Croton oil induced ear edema model	(B) Reduction of ear edema (63% inhibition at 400 mg applied topically)
Coutinho et al. (2012)	Flowers from *Kalanchoe pinnata* are a rich source of T cell-suppressive flavonoids	*In vitro* T cell proliferation assay in lymphnode cells isolated from mice	Aqueous flower extract, isolated flavonoids	(A) Effect on T cell mitogenesis	(A) Flower extract more active in inhibiting murine T cell mitogenesis than leaf extract (IC_50_ = 37.5 μg/mL vs 84.9 μg/mL)
				(B) Effect on cytokine production in lymph node cells	(B) All flavonoids inhibited murine T cell mitogenesis and IL-2 production, three out of five IL-4 production
Cruz et al. (2012)	*Kalanchoe pinnata* inhibits mast cell activation and prevents allergic airway disease	*In vitro* mast cell activation assay *In vivo* model of allergic airway disease in mice	Aqueous leaf extract and flavonoids quercetin and quercitrin	(A) Effect on mast cell activation and cytokine production	(A) Extract (250, 500 or 1,000 μg/mL) and quercetin (25, 50 or 100 μg/mL) led to dose-dependent decrease of mast cell degranulation; quercetin reduced IL-6 and TNF concentrations
				(B) Effect on OVA-induced airway hyperresponsiveness and airway inflammation	(B) treatment with extract (400 mg/kg) or quercetin (30 mg/kg) showed a reduction of airway reactivity; reduction in total cell count and especially in numbers of lymphocytes and eosinophils when treated with extract and quercetin; quercitrin had no effect
Braz et al. (2013)	Antiulcerogenic activity of aqueous extract from *Bryophyllum pinnatum* (Lam.) Kurz	*In vivo* antiulcerogenic rat model	Aqueous leaf extract	Effect on indomethacin-induced gastric ulcers	Extract (1 and 2 g/kg) inhibited 45.5% of the indomethacin-induced ulcer index
Chibli et al. (2014)	Anti-inflammatory effects of *Bryophyllum pinnatum* (Lam.) Oken ethanol extract in acute and chronic cutaneous inflammation	*In vivo* acute and chronic mice ear edema models induced by different irritant agents	Ethanolic leaf extract	Topical anti-inflammatory effects on mice ear edema induced by different agents (A) Croton oil	Extract (0.1, 0.5 and 1.0 mg/ear) inhibited ear edema induced by (A) Inhibition of 57%
				(B) Arachidonic acid	(B) Inhibition of 67%
				(C) Phenol	(C) Inhibition of 80%
				(D) Capsaicin	(D) Inhibition of 72%
				(E) Ethyl phenylpropiolate	(E) Inhibition of 75%
Ferreira et al. (2014)	Mechanisms underlying the antinociceptive, antiedematogenic, and anti-inflammatory activity of the main flavonoid from *Kalanchoe pinnata*	*In vivo* inflammation and pain mice model	Aqueous flowers extract (KPFE), ethyl acetate and butanol fraction, isolated flavonoid (KPFV)	(A) Effect on acetic acid-induced writhing	Results after s.c. application. (A) Inhibition of writhing (KPFE ID_50_ = 164.8 and KPFV 9.4 mg/kg)
				(B) Effect on Croton oil -induced ear edema	(B) Inhibition of ear edema (KPFE ID_50_ = 4.3 and KPFV = 0.8 mg/kg)
				(C) Effect on TNF-α concentration	(C) KPFE and KPFV reduced TNF-α concentration
				(D) Effect on COX-1 and COX-2 activity	(D) KPFV inhibited COX-1 (IC_50_ = 22.1 g/mL) and COX-2 (IC_50_ > 50 g/mL) activity
Tiwari (2015)	Comparative analysis of *Bauhinia tomentosa* L. and *Kalanchoe pinnata* Lam extracts with regard to their antinociceptive and antipyretic potentials in experimental animal models	*In vivo* inflammation and pain models in mice	Stem and root aqueous extract	(A) Anti-nociceptice effect in hot plate method	Results obtained at 200 and 400 mg/kg p.o. (A) Dose-dependent increase in latency time compared to control
				(B) Anti-nociceptive effect in acetic acid-induced writhing model	(B) Stem extract inhibited writhing response
				(C) Antipyretic effect in yeast induced hyperthermia	(C) Root extract had antipyretic effects
de Araújo et al. (2018)	Gastroprotective and antioxidant activity of *Kalanchoe brasiliensis* and *Kalanchoe pinnata* Leaf Juices against indomethacin and ethanol-induced gastric lesions in rats	*In vivo* acute gastric lesion rat models	Leaf press juice	(A) Effect in ethanol gastric lesion induction model	Results obtained at 250 mg/kg and 500 mg/kg p.o. (A) Dose dependent inhibition of up to 82%
				(B) Effect in indomethacin gastric lesions induction model	(B) Dose dependent inhibition of up to 63%
				(C) Effect in inflammatory cytokines in gastric tissue	(C) Reduction in IL-1β, TNF-α and NFκB levels
Indriyanti et al. (2018a)	Repairing effects of aqueous extract of *Kalanchoe pinnata* (lmk) pers. on lupus nephritis mice	*In vivo* lupus nephritis mice model/*in silico* identification of active compound	Aqueous leaf extract	(A) Effect on proteinuria	Results obtained at 200 mg/kg (A) Proteinuria level decreased to 30% in treatment groups
				(B) Identify active compound binding to glucocorticoid receptor *in silico*	(B) Bryophyllin A is the most active compound *in silico*
Indriyanti et al. (2018b)	T-cell activation controlling effects of ethyl acetate fraction of *Kalanchoe pinnata* (Lmk) pers on TMPD-treated lupus mice	*In vivo* lupus-like mice models	Ethyl acetate leaf fraction	Effect on leukocyte count	Total leukocytes reduced at 400 mg/kg
de Araújo et al. (2019)	Local anti-inflammatory activity: Topical formulation containing *Kalanchoe brasiliensis* and *Kalanchoe pinnata* leaf aqueous extract	*In vivo* paw and ear edema mice model	Aqueous leaf extract	(A) Local anti-inflammatory activity	(A) 5% extract showed statistically reduction of edema by 54%
				(B) Effect on IL-1β, and TNF-α levels	(B) 2.5% and 5% extract suppressed IL-1β and TNF-α levels
Naqvi et al. (2019)	Anti-platelet effect of *Bryophyllum pinnatum* aqueous extract in human blood	*In vitro* platelet aggregation assay	Aqueous leaf extract	Effect on platelet aggregation	Dose-independent anti-platelet effect on arachidonic acid and thrombin but not ADP
Pandurangan et al. (2019)	Evaluation of anti-inflammatory activity of *Bryophyllum calycinum* (Crassulaceae) on acute and chronic inflammation models	*In vivo* paw edema and granuloma mouse model	Whole plant ethanol/chloroform/n-hexane extracts	(A) Effect on Carrageenan-induced paw edema model	Results obtained at 400 mg/kg p.o. (A) Inhibition was observed for ethanol (92%), chloroform (88%) and n-hexane (86%) extracts
				(B) Effect on cotton pellet induced granuloma	(B) Ethanol extract showed 57% inhibitory effect in granuloma model
Andrade et al. (2020)	Anti-inflammatory and chemopreventive effects of *Bryophyllum pinnatum* (Lamarck) leaf extract in experimental colitis models in rodents	*In vivo* colitits rodent models	Hydroethanolic leaf extract	(A) Effect in 2.4-dinitrobenzene sulfonic acid (DNBS)-induced colitis in rats and in dextran sulfate sodium (DSS)-induced colitis in mice	Results obtained at 250 mg/kg and 500 mg/kg (A) chemopreventive and anti-inflammatory effects and reduction in disease activity index score
				(B) *In vitro* anti-inflammatory effects	(B) downregulation of toll-like receptor, IL-1β, TNF-α and NFκB
Latif et al. (2020)	Phytochemical analysis and *in vitro* investigation of anti-inflammatory and xanthine oxidase inhibition potential of root extracts of *Bryophyllum pinnatum*	*In vitro* anti-inflammatory investigation	Aqueous and methanolic root extract	(A) Anti-inflammatory activity (protein denaturation)	(A) Aqueous extract had an IC_50_ value of 570 μg/mL
				(B) Effect on xanthine oxidase inhibition	(B) Methanol extract most effective
Lourenço et al. (2020)	Identification of a selective PDE4B inhibitor from *Bryophyllum pinnatum* by target fishing study and *in vitro* evaluation of quercetin 3-O-α-L-arabinopyranosyl-(1→2)-O-α-L-rhamnopyranoside	*In silico* target fishing and *In vitro* enzyme inhibition assay	Single compound quercetin 3-O-a-L arabinopyranosyl-(1→2)-O-a-L-rhamnopyranoside	(A) Target fishing	(A) Anti-inflammatory activity explained by inhibition of PDE4B *in silico*
				(B) Inhibition of PDE4B by compound	(B) *In vitro* experiments showed highly selective inhibition of PDE4B by compound (10 μM)
Coutinho et al. (2021)	Wound healing cream formulated with *Kalanchoe pinnata* major flavonoid is as effective as the aqueous leaf extract cream in a rat model of excisional wound	*In vivo* excisional wound rat model	Aqueous leaf extract	Wound healing effect	Results obtained at 6% topical application
					On day 12, wounds treated with extract cream showed 95% healing (compared to control 76% healing); better reepithelization and denser collagen fibers
Dantara et al. (2021)	Effect of *Bryophyllum pinnatum* leaves ethanol extract in TNF-α and TGF-β as candidate therapy of SLE in pristane-induced sle balb/c mice model	*In vivo* lupus mice model	Ethanol leaf extract	Effect on inflammation markers in pristane-induced lupus mice	Results obtained at 10.5–42 mg/kg/d i.p
					Percentages of maturation of B cells and TNF-α were decreased; percentages of TGF-β were increased (anti-inflammatory agent)
de Araújo et al. (2021)	Gastric ulcer healing property of *Bryophyllum pinnatum* leaf Extract in chronic model *in vivo* and gastroprotective activity of its major flavonoid	*In vivo* gastric lesion rodent models	Aqueous leaf extract and isolated flavonoid (quercetin 3-O-α-L-arabinopyranosyl-(1→2)-O-α-L-rhamnopyranoside)	(A) Ulcer healing properties of leaf extract in acetic-acid induced chronic ulcer model	(A) Treatment with the extract at 250 and 500 mg/kg stimulated the healing process (76% and 81% inhibition, respectively)
				(B) Gastroprotective effects of isolated flavonoid in gastric lesions induced by ethanol and indomethacin models	(B) 5 mg/kg of isolated compound reduced ethanol-induced lesion by 49% and indomethacin-induced lesion by 51%
				(C) *In vitro* effect in acetic acid-induced chronic gastric ulcer model	(C) Downregulation of IL1-β, TNF-α, expression of COX-2 and NF-κB (p65) (250 and 500 mg/kg)
Morais Fernandes et al. (2021)	*Bryophyllum pinnatum* markers: CPC isolation, simultaneous quantification by a validated UPLC-DAD method and biological evaluations	*In vitro* model on xanthine oxidase activity	Hydroethanolic leaf extract and isolated compounds	Effect on xanthine oxidase activity	Inhibitory effect on xanthine oxidase with IC_50_ values of >220 μg/mL (extract), 168 μM (kaempferol 3-O-α-L-arabinopyranosyl-(1→2)-O-α-L-rhamnopyranoside), 124 μM (quercetin 3-O-α-L-rhamnopyranoside)
Santos et al. (2021)	*Bryophyllum pinnatum* compounds inhibit oxytocin-induced signaling pathways in human myometrial cells	*In vitro* assay on oxytocin induced activation of MAPKs in human myometrial cells	Leaf press juice and fractions	Effect on phosphorylation of MAPKs in human myometrial cells	Press juice (20 mg/mL) inhibited oxytocin-driven activation of MAPKS JNK/SAPK and ERK1/2 as did the bufadienolide-enriched fraction (2.2 μg/mL) by 50%
Singh et al. (2022)	Comparative evaluation of anti-arthritic activity of *Pongamia pinnata*, *Bryophyllum pinnata* and their combined formulation in FCA induced arthritis rat model	*In vivo* arthritis rat model	Ethanolic leaf extract	(A) Effect on arthritic score	(A) 500 mg/kg led to more than 40% reduction of arthritic score (B) 500 mg/kg led to 2.4 times longer pain response
				(B) Anti-nociceptive activity in hot plate method	
de Araújo et al. (2023)	Gel formulated with *Bryophyllum pinnatum* leaf extract promotes skin wound healing *in vivo* by increasing VEGF expression: A novel potential active ingredient for pharmaceuticals	*In vivo* skin wound rat model	Aqueous leaf extract, topical gel 5%	(A) Wound healing effect on induced skin wound in rats	(A) Topical gel led to 60% reduction of wound area after 14 days compared to control
				(B) Anti-inflammatory effect on wound tissue	(B) Reduction of IL1β and TNF-α
				(C) Effect on angiogenesis estimated by VEGF expression	(C) Increased expression of VEGF

**TABLE 2:** Methodological characteristics, plant material, main results of included publications on anti-contractility and smooth muscle relaxing effects of *B. pinnatum.*

Reference	Title	Type of study	Bryophyllum pinnatum material	Main outcome assessment	Main results
Gwehenberger et al. (2004)	Effect of *Bryophyllum pinnatum versus* fenoterol on uterine contractility	*Ex vivo* organ bath model with (human) myometrial biopsies from term caesarean section compared to fenoterol	Leaf press juice and 5% leaf press juice solution for i.v. injection	(A) Effect on spontaneous contractions	(A) Concentration-dependent reduction in area under the curve (AUC; maximal by 16% at 10^4^ mg/L); increase in contraction frequency
				(B) Effect on OT-induced contraction	(B) Maximal reduction of AUC by 20% at 5 × 10^3^ mg/L; decreased contraction frequency at 5 × 10^3^ mg/L
Mans et al. (2004)	Assessment of eight popularly used plant-derived preparations for their spasmolytic potential using the isolated guinea pig ileum	*Ex vivo* organ bath model on ability to reduce the strength of smooth muscle contraction in guinea pig ileum	Aqueous leaf extract	(A) Effect on contraction induced by acetylcholine	(A) Inhibitory effect on acetylcholine-induced contractions only observed at 10 mg/mL
				(B) Effect on contraction induced by histamine	(B) Dose dependent inhibitory effect on contraction force induced by histamine (40%–95% at doses from 0.01 to 10 mg/mL)
Plangger et al. (2006)	Intravenous tocolysis with *Bryophyllum pinnatum* is better tolerated than beta-agonist application	Retrospective matched-pair study on tocolytic effect of i.v. infused *B. pinnatum* compared to beta-agonist (n = 134)	5% leaf press juice solution for i.v. injection	(A) Prolongation of pregnancy	(A) Equal in the prolongation of pregnancy and (B) Gestational age at delivery
				(B) Gestational age at delivery	
				(C) Maternal tolerability	(C) Fewer adverse effect in the Bryophyllum group (*p* = 0.02^a^)
Ozolua et al. (2010)	Effects of aqueous leaf extract of *Bryophyllum pinnatum* on guinea pig tracheal ring contractility	*Ex vivo* organ bath model on effect on contractile responses of isolated guinea pig tracheal rings	Aqueous leaf extract	(A) Effect on histamine-induced contraction	0.25–1.0 mg/mL of the extract in organ baths significantly reduced the maximal contractile responses induced by (A) histamine and (B) carbachol
				(B) Effect on carbachol-induced contraction	
Simões-Wüst et al. (2010)	Juice of *Bryophyllum pinnatum* (Lam.) inhibits oxytocin-induced increase of the intracellular calcium concentration in human myometrial cells	*In vitro* intracellular free calcium assay in human myometrial cell line	Leaf press juice	Effect on OT-induced increase of intracellular calcium in human myometrial cells	Press juice prevented the OT-induced increase in [Ca^2+^]_i_ in hTERT-C3 human myometrial cells in a dose-dependent manner, achieving a ca. 80% inhibition at a 2% concentration
Wächter et al. (2011)	Leaf press juice from *Bryophyllum pinnatum* (Lamarck) Oken induces myometrial relaxation	*Ex vivo* organ bath model with myometrial biopsies from term cesarean section	Leaf press juice and MPLC fractions	(A) Effect on amplitude of contraction	(A) Amplitude reduced to 78% of baseline after second addition of 2 μL press juice; reduced to 70% after second addition of a fraction tentatively assigned to flavonoids
				(B) Effect on AUC of contraction	(B) AUC reduced to 82% after first addition of 2 μL press juice; reduced to 51% after first addition of fraction tentatively assigned to flavonoids
Schuler et al. (2012)	*Bryophyllum pinnatum* inhibits detrusor contractility in porcine bladder strips - A pharmacological study towards a new treatment option of overactive bladder	*Ex vivo* organ bath model porcine detrusor strips compared to oxybutynin	Leaf press juice	(A) Inhibitory effect measurements with electrical field stimulation (B) Relaxant effect measurements on carbachol pre-contracted strips	(A) Press juice 5% inhibited electrically induced contractions by 75% relative to time-matched control (B) Press juice 10% maximum relaxant effect on carbachol re-contracted strips was 19%
Betschart et al. (2013)	Randomized, double-blind placebo-controlled trial with *Bryophyllum pinnatum versus* placebo for the treatment of overactive bladder in postmenopausal women	RCT vs placebo (n = 20)	Leaf press juice tablets 50%	(A) Reduction of micturition frequency per 24 h	(A) Trend in reduction of micturition frequency per 24 h from 9.5 to 7.8
				(B) Quality of life	(*p* = 0.064)
					(B) Improvement of quality of life did not differ between the two groups
Fürer et al. (2015a)	Inhibition of porcine detrusor contractility by the flavonoid fraction of *Bryophyllum pinnatum*--a potential phytotherapeutic drug for the treatment of the overactive bladder syndrome	*Ex vivo* organ bath model measuring repeated electrically induced contractions of porcine detrusor strips	Leave press juice and fractions prepared from methanolic leaf extract	(A) Effect of press juice	(A) After initial stimulation, press juice 10% led to a reduction of detrusor contractility to 59%
				(B) Effect of a fraction enriched in flavonoids	(B) After initial stimulation, a fraction enriched in flavonoids showed a significant reduction of the contractility to 21% at a concentration of 1 mg/mL
				(C) Effect of a fraction enriched in bufadienolides	(C) A fraction enriched in bufadienolides had stimulatory effects (max. concentration tested 40 μg/mL)
Mans et al. (2015)	Evaluation of surinamese medicinal plants for their potential bronchospasmolytic effects in isolated guinea pig tracheal chains	*Ex vivo* organ bath model on effect on contractile responses of isolated guinea pig tracheal rings	Aqueous leaf extract	(A) Effect on acetylcholin-induced contraction	Aqueous extract (10 mg/mL) reduced the force of contraction of the tracheal chains caused by both (A) acetylcholine and (B) histamine by 40%–70%
				(B) Effect on histamine-induced contraction	
Bachmann et al. (2017)	Potential of *Bryophyllum pinnatum* as a detrusor relaxant: an *in vitro* exploratory study	*Ex vivo* organ bath model measuring KCl-induced contractility of porcine detrusor strips	Leaf press juice, fractions enriched in flavonoids or bufadienolides, flavonoid aglycon mix	(A) Effect of leaf press juice on contraction force	(A) Press juice increased the contraction force
				(B) Effect of bufadienolide- and (C) Flavonoide-enriched fractions on contractions force	(B) A purified bufadienolide-enriched fraction (0.1–1.0 mg/mL) led to significant inhibition of detrusor contractility
					(C) A flavonoid-enriched fraction did not affect contraction force and flavonoid aglycons (0.15–0.5 mg/mL) led to a concentration-dependent lowering of the contraction force
Santos et al. (2018)	A bufadienolide-enriched fraction of *Bryophyllum pinnatum* inhibits human myometrial contractility *in vitro*	*Ex vivo* organ bath model with myometrial biopsies from term cesarean section	Leaf press juice, fractions enriched in flavonoids or bufadienolides, flavonoid aglycon mix	(A) Effect of leaf press juice on contraction strength	(A) 10 mg/mL of leave press juice
				(B) Effect of bufadienolide- and (C) Flavonoide-enriched fractions and aglycon mix on contractions strength	(B) 1 μg/mL bufadienolide-enriched fraction
					(C) 150 μg/mL of flavonoid-enriched fraction and 6.2 μg/mL of flavonoid aglycon mixture lead to reduction of contraction strength of about 40%
Simões-Wüst et al. (2018)	Two randomised clinical trials on the use of *Bryophyllum pinnatum* in preterm labor: results after early discontinuation	Trial I: RCT vs placebo (double blind; n = 26) Trial II: RCT vs nifedipine (open-label; n = 27)	Trial I: ethanolic tincture 33%	(A) Trial I: prophylaxis of preterm labor in patients at risk	(A) and (B) Trials discontinued early due to slow patient recruitments, data not sufficient for concluisions on efficacy
			Trial II: leaf press juice tablets 50%	(B) Difference in patient response to tocolysis	(C) Overall a good tolerability was observed
				(C) Tolerability	
Santos et al. (2019)	*Bryophyllum pinnatum* enhances the inhibitory effect of atosiban and nifedipine on human myometrial contractility: An *in vitro* study	*Ex vivo* organ bath model with (human) myometrial biopsies from term cesarean section viability assays in human myometrial cells	Leaf press juice	(A) Effect on contraction strength in combination with standard tocolytics	(A) Press juice plus atosiban promoted a decrease to 49%; press juice and atosiban alone lowered it to 71% and 81%, respectively. Press juice plus nifedipine decreased strength to 40%, press juice and nifedipine alone lowered it to 79% and 71% (B) Test substances showed no effect on cell viability
				(B) Cell toxicity	
Santos et al. (2021)	*Bryophyllum pinnatum* compounds inhibit oxytocin-induced signaling pathways in human myometrial cells	*In vitro* cell signaling assays in human myometrial cells	Leaf press juice, fractions enriched in flavonoids or bufadienolides, flavonoid aglycon mix	(A) Effect on OT-stimulated intracellular calcium increase	(A) Concentration-dependent decrease of OT-induced increase of intracellular free calcium concentration was observed for all test substances but none of them was as strong as press juice (inhibition of 70%); significant inhibition was obtained with 4.335 μg/mL of flavonoid fraction and 0.055 μg/mL bufadienolide fraction (B) inhibition of OT-driven activation of MAPKs ERK1/2 and SAPK/JNK by press juice and bufadienolide-enriched fraction
				(B) Effect on OT-driven activation of MAPKs	
Mirzayeva et al. (2023)	*Bryophyllum pinnatum* and improvement of nocturia and sleep quality in women: a multicentre, nonrandomized prospective trial	Multicenter, nonrandomized prospective clinical trial (n = 49)	Leaf press juice tablets 50%	(A) Nocturia (voids per night)	(A) Nocturia diminished from 3.2 to 2.3 voids per night (*p* < 0.001)
				(B) Sleep disorders (PSQI score)	(B) PSQI score decreased from 7.7 to 6.6 (*p* = 0.004)

^a^ Only in case of clinical studies *p*-values are shown.

#### 3.1.2 Anti-nociceptive and anti-inflammatory effects

Neither retrospective nor prospective clinical studies were encountered. Indications for clinical evidence consist of reported use in ethnomedicine (cf. Introduction) and have not been included in the present review. *In vivo* and *in vitro* studies cover a wide range of pathophysiological topics related to inflammatory and nociceptive processes and are described in the following.

The analgesic effect of *B. pinnatum* has mainly been investigated using animal models (mice and rats), where thermal (hot plate method) and chemical (e.g., acetic acid) nociceptive stimuli are used to assess changes in pain level. Several publications reported a dose-dependent analgesic effect for *B. pinnatum* extract in the chemically-induced writhing model (Olajide et al., 1998; Pal et al., 1999; Igwe and Akunyili, 2005; Ojewole, 2005). Further, an increase in pain threshold using the hot plate model was observed (Igwe and Akunyili, 2005; Singh et al., 2022). In two publications, *B. pinnatum* stem extract and an isolated steroidal derivative (urs stigmast-4, 20 (21), 23-trien-3-one) also led to a reduction in acetic-acid induced writhing ((Afzal et al., 2012; Tiwari, 2015), respectively).

An often used *in vivo* set up to assess the systemic anti-inflammatory effect of *B. pinnatum* is the paw or ear edema model. In this, edema is induced by various substances administered in the ear or paw of the test animal, and inflammation is assessed by measuring weight or volume of the body part. *B. pinnatum* leaf extracts inhibited inflammation induced by carrageen (Olajide et al., 1998; Pandurangan et al., 2019) and formaldehyde (Gupta et al., 2010). Moreover, an extract from *B. pinnatum* stem reduced croton oil-induced edema in rat ear (Chaturvedi et al., 2012). Also, after topical application, another *B. pinnatum* extract was able to inhibit croton oil-, arachidonic acid-, phenol- and capsaicin-induced ear edema in mice, with histopathologic evaluation showing reduced tissue infiltration by inflammatory cells (Chibli et al., 2014). In one publication, the inhibitory effect of a flower extract of *B. pinnatum* on croton-oil induced ear edema was shown (Ferreira et al., 2014). Quercetin 3-O-α-L-arabinopyranosyl (1→2) α-L-rhamnopyranoside, the most abundant flavonoid in *B. pinnatum* leaves and flowers, was shown to have anti-inflammatory and anti-nociceptive effects *in vivo* (Coutinho et al., 2012; Ferreira et al., 2014). This flavonoid with a rather unusual glycosylation pattern was further shown to inhibit the activity of COX-1 as well as COX-2 enzymes (Ferreira et al., 2014).

Local administration of an aqueous extract of *B. pinnatum* resulted in the reduction of inflammation in the ear and paw edema model (de Araújo et al., 2019). This led to investigations regarding wound healing properties of *B. pinnatum*, since the treatment of wounds is also a frequent use in ethnomedicine. The topical application of a cream containing an aqueous leaf extract and another containing the major flavonoid (quercetin 3-O-a-L-arabinopyranosyl-(1→2)-a-L-rhamnopyranoside) showed better re-epithelization and denser collagen after 12 days of application in a rat excision model (Coutinho et al., 2021). After showing that *B. pinnatum* applied via a gel formulation achieved wound healing properties by reducing wound area, authors of a recent publication also aimed to find the mechanism of action. They found out that besides reducing inflammatory infiltrate in the wound area, angiogenesis was also improved based on increased expression of vascular endothelial growth factor (VEGF) (de Araújo et al., 2023).

In an *in vivo* acute gastric lesion induction model, *B. pinnatum* press juice (de Araújo et al., 2019) and a methanolic extract (Pal and Nag Chaudhuri, 1991) showed gastro protective and ulcer-healing effects, and inhibited inflammatory reaction by reducing the levels of interleukin 1β (IL-1β), tumor necrosisfactor α (TNF-α) and the expression of nuclear factor κ-light-chain-enhancer of activated B cells (NF-κB-p65) (de Araújo et al., 2018; de Araújo et al., 2021). In an experimental study focusing on the use of *B. pinnatum* for treatment of Colitis ulcerosa and Crohn’s disease, hydroethanolic leaf extract showed downregulation of toll-like receptor and NF-κB-p65 *in vivo* (rats and mice), improved cytoarchitecture of colon tissue, as well as mucosa protection, and *in vitro* reduction in pro-inflammatory mediators (Andrade et al., 2020).

Some identified publications focused on the immunomodulatory effect of *B. pinnatum* extracts and compounds. In lupus mice, *B. pinnatum* reduced T cell activation (Indriyanti et al., 2018b) and B cell maturation, decreased production of TNF-α (Dantara et al., 2021) and improved histopathological nephritis markers (Indriyanti et al., 2018a). In addition, the aqueous extract and the flavonoid quercetin reduced *in vitro* mast cell activation and *in vivo* airway inflammation in allergic airway models (Cruz et al., 2012).

In myometrial cells, oxytocin (OT) is known to trigger inflammatory signaling pathways by leading to activation of mitogen activated protein kinases (MAPK) stress-activated protein kinase or c-jun N-terminal (JNK/SAPK) and extracellular-signal regulated kinases (ERK1/2), which are known to influence the expression of many downstream enzymes such as COX-2, and play important roles in calcium-independent contractility regulation. Data on substances from *B. pinnatum* leaves revealed that press juice, a flavonoide-enriched fraction and a bufadienolide-enriched fraction inhibited OT-induced JNK/SAPK and ERK1/2 activation by phosphorylation (see (Santos et al., 2021) and references therein). Further, the effects of *B. pinnatum* extracts on various enzymes involved in inflammatory processes were investigated. In these studies, leaf extract as well as root extract inhibited xanthinoxidase (Latif et al., 2020; Morais Fernandes et al., 2021). 3-O-α-L-Arabinopyranosyl-(1→ 2)-O-α-L-rhamnopyranoside was shown to inhibit phosphodiesterase 4 (PDE4) (Lourenço et al., 2020) and the antiplatelet effect of the aqueous extract indicated an effect on the arachidonic acid pathway (as does the inhibition of COX) (Naqvi et al., 2019).

#### 3.1.3 Relaxant/contractility-inhibitory effects

Clinical evidence for the use of *B. pinnatum* in tocolysis can be found in a retrospective matched-pair study from 2006. It was shown that pregnancy prolongation under treatment with *B. pinnatum* was comparable to beta-agonists, but with notably fewer side effects (Plangger et al., 2006). An effort was made to gain data from randomized clinical trials; however, the two initiated trials had to be stopped early due to difficulties in patient recruitment. Both trials showed good tolerability of *B. pinnatum* although data were not sufficient to support statements on clinical efficacy (Simões-Wüst et al., 2018). The use of *B. pinnatum* in preterm labor management has been reported since the 1970s, but corresponding studies were not identified in the present review since they were published exclusively in German. The latter consisted of an initial retrospective analysis showing that *B. pinnatum* was as effective as fenoterol, the standard treatment at that time, and that the dosage of fenoterol could be reduced when combined with *B. pinnatum* preparations (Hassauer et al., 1985). These results were supported by comparable retrospective analyses conducted in the following years (Daub, 1988; Vilàghy, 2002).

Two clinical trials concerned the use of *B. pinnatum* in women with overactive bladder syndrome (one pilot, double-blind and placebo-controlled, the other prospective but not randomized). In the case of the pilot study, a trend in reduction of micturition frequency and a positive trend for efficacy were apparent (Betschart et al., 2013). In the before-after comparison, a reduction in nocturia and a beneficial effect on sleep quality were observed (Mirzayeva et al., 2023).

The use of *B. pinnatum* preparations as a tocolytic, as mentioned above, is supported by experimental evidence. The first experimental data on the effect of *B. pinnatum* on myometrial contractility were published in 2004. In an *ex vivo* organ-bath model, myometrium biopsies gained from women undergoing term caesarean section were used to assess the relaxing effect of *B. pinnatum* aqueous leaf extract, and the effect was compared to the β-agonist fenoterol. The results showed that *B. pinnatum* reduced the strength of contractions, which was confirmed by later work and supporting its use as a tocolytic (Gwehenberger et al., 2004; Wächter et al., 2011; Santos et al., 2019). To gain more information on the mode of action, the effect of *B. pinnatum* (press juice) on the OT-triggered free intracellular calcium [Ca^2+^]_i_ increase was thereafter examined on a cellular level. This was done *in vitro* using a human myometrial cell line obtained by transformation from the uterus of a (non-pregnant) woman undergoing hysterectomy (hTERT-C3 cells). Data showed a concentration-dependent inhibition of [Ca^2+^]_i_ increase (Simões-Wüst et al., 2010). More recently, the effect of *B. pinnatum* press juice was investigated using an additional human myometrial cell line obtained from a pregnant uterus (PHM1-41 cells) and the inhibitory effect on the [Ca^2+^]_i_ increase could be confirmed (Santos et al., 2021). In an attempt to determine which secondary plant compounds are responsible for the OT-induced inhibition of myometrial contractility, the organ-bath model was used again to assess the effects of a bufadienolide- and a flavonoid-enriched fraction. All test substances led to a decrease in myometrial contractility, but the bufadienolide-enriched fraction was most effective (Santos et al., 2018). Both fractions also inhibited the OT-driven increase of [Ca^2+^]_i_ in myometrial cells *in vitro* and most interestingly, appeared to have a synergistic effect (Santos et al., 2021).

Besides myometrial tissues, other smooth muscle types were tested with similar approaches using the organ bath model to investigate the relaxant effect of *B. pinnatum*. In porcine bladder strips (detrusor muscle) *B. pinnatum* press juice and a flavonoid-enriched fraction inhibited electrically induced contraction and also carbachol pre-contracted strips showed reduced contractility (Schuler et al., 2012; Fürer et al., 2015a), whereby a dose-independent initial increase in contractility was observed in one study (Fürer et al., 2015a). In a later study where porcine detrusor muscle strips were stimulated by potassium chloride a highly purified bufadienolide-enriched fraction was the most effective and led to a concentration-dependent lowering of the contraction force. *B. pinnatum* press juice alone however increased the contraction force of muscle strips (Bachmann et al., 2017). When comparing eight different plants known to be used owing to their spasmolytic potential to reduce the histamine-induced contracting force of guinea pig ileum, a *B. pinnatum* leaf extract was able to inhibit it progressively from 40% to 95% (Mans et al., 2004). Finally, *ex vivo* experiments based on an organ bath model revealed that *B. pinnatum* extract reduces the contractility of tracheal rings of guinea pigs (Ozolua et al., 2010; Mans et al., 2015). These latter experiments are in line with the already mentioned use of *B. pinnatum* in ethnomedicine for inflammatory gastrointestinal diseases and as a cough remedy, respectively.

### 3.2 Case series

In this consecutive case series, five patients suffering from dysmenorrhea were treated with Bryophyllum 50% chewable tablets over 3–6 months. All five patients (aged 14–27 years) reported a reduction in pain symptoms (shown by a reduction of the NRS score by at least 2) and 4 out of 5 indicated a reduced intake of painkillers during menstruation. None of the patients mentioned adverse reactions. Cases are described in more detail below and a summary of patients and outcomes can be found in Table 3.

**TABLE 3:** Patient characteristics and objective outcomes before starting treatment with Bryophyllum 50% tablets and thereafter.

Case number	Age (years)	Age at menarche (years)	Additional anamnesis	Bryophyllum 50% treatment plan	Treatment duration till follow up (months)	NRS score		Pain medication intake	
						Before	After	Before	After
1	18	14	Migraine	3—0—3	3	7	3–4	2 × 400 mg ibuprofen for first 2 days of cycle	sporadically
2	27	11	Mammary cyst	2—2—2	3	6	3	1 × 400 mg ibuprofen on first day of cycle	1 × 400 mg ibuprofen
3	21		Smoking	2—2—2 From 16th day of cycle	6	10	≤6	2 × 400 mg ibuprofen for 2 days	0
4	20	13	-	2—2—2 From 16th day of cycle	3	3–5	≤3	500 mg paracetamol if needed	0
5	14		-	2—2—2	3	8	6	Yes but not specified	Only occasionally necessary

#### 3.2.1 Patient 1

An 18-year-old virgin patient presented with dysmenorrhea and hypermenorrhea. She complained about menstruation-associated migraine. The patient had had her menarche when she was 14-years old; family-history revealed only migraine on the paternal side. She did not lose workdays during menstruation, but on the first and second period days, she reported an NRS score of 7. Her usual analgesic requirement per period was 2 times ibuprofen 400 mg per day for 2 days. Abdominal examination was inconspicuous, transabdominal ultrasound scan was difficult with empty bladder, but a uterus malformation was suspected (European society of gynecological endoscopy U2a dd U3a), both ovaries (and both kidneys) unremarkable, no evidence of endometriosis. Iron levels were determined (no iron deficiency). The patient started treatment with Bryophyllum 50% chewable tablets (3-0–3).

Three months after start of the Bryophyllum 50% chewable tablets treatment, at follow-up, the patient mentioned poor compliance during the first 30 days, but regular use thereafter with strong improvement in symptoms. Her NRS score was reduced to 3-4, she used ibuprofen during menstruation only sporadically and perceived a subjective improvement in migraine. Transabdominal ultrasound examination confirms previous findings.

#### 3.2.2 Patient 2

A 27-year-old woman presented with primary dysmenorrhea on the first and second days of her period. She reported an NRS score of 6, and subjective hypermenorrhea and analgesic treatment on the first day of her period (1x ibuprofen 400 mg), but no lost work days during menstruation. At anamnesis, she reported menarche at 11 years of age, a known mammary cyst on the left side (US 2021: breast imaging reporting and data system 2), never being pregnant and an in conspicuous family anamnesis. As contraception, she uses condoms; vaginal and transvaginal ultrasound examinations inconspicuous. The patient started treatment with Bryophyllum 50% chewable tablets (2-2-2).

Three months later, at follow-up, the patient reported an NRS score of 3 and commented “Before the treatment, the pain woke me up in the night, now I hardly know that I have my period”. However, she was still taking one ibuprofen 400 mg tablet on the first day of her period; “I take it preventively” she said.

#### 3.2.3 Patient 3

A 21-year-old woman presented with primary dysmenorrhea and a desire to have children. Gynecological examination was unremarkable. Apart from smoking (10 cigarettes/day) she was otherwise healthy; family anamnesis inconspicuous. Current complaints comprised a severe dysmenorrhea-related NRS score of 10, requiring analgesics (ibuprofen 400 mg 2 times per day for the first 2 days of the period). Patient was advised to take Bryophyllum 50% chewable tablets 3x daily (2-2-2) from the 16th day of the cycle until the end of menstruation; in addition, the patient had to start taking folic acid.

Six months later, the patient presented with a positive pregnancy test (pregnancy week 5 and 3 days). A transvaginal ultrasound revealed a uterus with gestational sac 7.2 × 4.5 mm and yolk sac 1.3 mm, no embryo visible yet. When asked about the effect of Bryophyllum 50% chewable tablets on dysmenorrhea, she reported that the NRS score was 6 or even lower with no need for analgesics any longer.

#### 3.2.4 Patient 4

A 20-year-old woman presented with dysmenorrhea that lasted for approximately 2 years and a desire to have children for the last approximately 6 months. Vaginal examination was unremarkable, but transvaginal ultrasound revealed a retroflected uterus, an endocervical polyp 11.7 × 3.9 mm and a myoma of 8.4 × 8.8 mm. Endometrium was 12 mm secretive and both ovaries were unremarkable. The patient had had her menarche when she was 13 years old and is otherwise healthy; family anamneses inconspicuous. Current complaints comprised a dysmenorrhea-related NRS score of 3-5, and a partial analgesic requirement (paracetamol 500 mg if needed). Patient was advised to take Bryophyllum 50% chewable tablets 3x daily (2-2-2) from the 16th day of the cycle until the end of menstruation; in addition, she had to start taking folic acid.

Three months later the patient presented with a positive pregnancy test (pregnancy week 5 and 4 days). A transvaginal ultrasound revealed a uterus with gestational sac 11.1 × 6.2 mm and yolk sac 1.1 mm, no embryo visible yet; intracervical intramyometrical anterior wall myoma stable, no polyp could be visualized; both ovaries were inconspicuous. Asked about the effect of Bryophyllum 50% chewable tablets on dysmenorrhea (two cycles), she reported that her NRS score was 3 or even lower; no need for analgesics. She should continue taking 2 tablets 3x daily (2-2-2; for supporting pregnancy), and come, and will come for a check-up in 2 weeks. She delivered a (healthy) child 8 months later by secondary caesarean section (upon suspicious cardiotocogram), without complications.

#### 3.2.5 Patient 5

A 14-year-old female patient presented with hypermenorrhea on day 1 and 2 of menstrual cycle (duration of menses 3–4 days) and severe dysmenorrhea on the first day of menstruation (reporting an NRS score of 8). She reported that an intake of analgesics (not specified) was always necessary on the day in question. She further reported that on these days she was sometimes unable to attend school and was prevented from pursuing other activities. Patient was prescribed Bryophyllum 50% chewable tablets 3 times a day 2 tablets (2-2-2).

After 3 months of following the procedure, patient presented with an NRS score of 6 on first day of menstruation and reported a reduced intake of analgesics (only occasionally necessary). Furthermore, she was no longer prevented from carrying out everyday activities.

## 4 Discussion

Taken together, the pathophysiology of dysmenorrhea syndrome points towards inflammatory events, uterine spasms and vasoconstriction, as the three pillars of dysmenorrhea symptomatology (see Introduction). The reviewed *in vitro, in vivo* and clinical data suggest that at least anti-nociceptive/anti-inflammatory effects and uterine relaxation could be promoted by *B. pinnatum* preparations. In the following, the evidence most relevant for estimating potential effects of *B. pinnatum* on dysmenorrhea-related pathophysiological processes is discussed.

A variety of studies demonstrated the analgesic effect of *B. pinnatum* extracts and compounds (Olajide et al., 1998; Pal et al., 1999; Igwe and Akunyili, 2005; Ojewole, 2005), explaining its use for pain treatment in ethnopharmacy and suggesting that they could attenuate dysmenorrhea-associated pain. Moreover, the reported anti-inflammatory effects of *B. pinnatum* make it an interesting candidate for the treatment of dysmenorrhea, since inflammatory events are an important part of its pathophysiology. Besides the data on systemic anti-inflammatory effects shown in various rodent models (Olajide et al., 1998; Gupta et al., 2010; Chibli et al., 2014; Pandurangan et al., 2019), the inhibitory effects on specific regulators of the inflammatory cascade are of special interest. Leaf juice and extracts inhibited the activation of NF-κB-p65, MAPKs JNK/SAPK and ERK1/2, and the activity of the downstream enzymes COX-1 and COX-2 (Ferreira et al., 2014; de Araújo et al., 2018; Andrade et al., 2020; Santos et al., 2021). Quercetin 3-O-α-L-arabinopyranosyl (1→2) α-L-rhamnopyranoside showed to be at least partly responsible for the inhibition of COX-1 and COX-2 (Ferreira et al., 2014).

Further rational for the exploration of *B. pinnatum* as a new treatment option for dysmenorrhea is given by the identified publications regarding the smooth muscle relaxing effect observed in *in vitro* and *ex vivo* studies in the context of other gynecological as well as non-gynecological indications like preterm labor, overactive bladder syndrome, and gastrointestinal disease. Especially in the pregnant myometrium, the hormone OT plays a major role in the induction of contractions. *In vitro* research related to the use of *B. pinnatum* as a tocolytic showed the direct relaxation of myometrial muscle strips, and the inhibition of OT-triggered calcium-signaling in myometrial cell lines from both pregnant and non-pregnant uterus (Gwehenberger et al., 2004; Simões-Wüst et al., 2010; Santos et al., 2018; Santos et al., 2019; Santos et al., 2021).

In this context, the research efforts on an arginine-vasopressin (AVP) related approach for treatment of dysmenorrhea are worth mentioning. The approach is based on the high secretion of AVP in the menstruating uterus, where this hormone is a relevant factor for myometrial contractility and decreased uterine blood flow (Åkerlund et al., 1979; Strömberg et al., 1984; Arrowsmith, 2020). Furthermore, the approach has to do with the high expression of the AVP receptor 1a (AVPR1a) in the non-pregnant myometrium [approximately five times higher than the very similar OT receptor (OTR) (Åkerlund, 2002)]. Therefore, AVPR1a antagonists could be a potential new treatment option for primary dysmenorrhea. One example of an intensively studied AVPR1a-antagonist is relcovaptan, which was investigated in a prospective clinical study with non-pregnant women. The administration of relcovaptan preceding a bolus injection of AVP or OT showed a dose related reduction in intrauterine pressure after AVP, but not after OT application (Steinwall et al., 2004). While this supports AVPR1a as a therapeutic target, adverse reactions (e.g., headaches, nausea and hypotension) might limit future application (Lemmens-Gruber, 2006). Whether the inhibitory effect of *B. pinnatum* on myometrial contractility is mediated through inhibition of the OTR or also AVPR1a, is currently unknown. Due to the high receptor homology of OTR and AVPR1a and the extensive cross talk between OT and AVP (Åkerlund et al., 1999; Arrowsmith, 2020), it is conceivable that *B. pinnatum* extracts also inhibit AVP-mediated signaling. Which contractility-associated pathways *B. pinnatum* press juice inhibits and, most importantly, whether it only inhibits OT-induced signaling or, in addition, AVP-induced increase of [Ca^2+^]_i_ in myometrial cells deserves further investigations.

The effectiveness observed in the first five patients treated supports our suggestion to repurpose *B. pinnatum* for the treatment of dysmenorrhea: in all five women, the NRS score decreased by at least 2 (and up to 4) and 4 out of 5 patients reported a reduced intake of pain medication during menstruation (Table 3). Case series can be seen as a low-cost and feasible method for a preliminary proof of concept for a certain treatment in a real-world setting. However, results have to be interpreted with caution, given the low number of patients involved and the absence of a placebo-control group. In our case, this might be particularly relevant as previous studies have revealed that patients with primary dysmenorrhea respond favorably to placebo. In a study from 1989, a third of patients treated with placebo experienced symptom improvement in the first treatment cycle. The study was conducted over four consecutive cycles over which the efficacy of placebo treatment decreased continuously, with only 10% of patients still reporting an effect in the fourth cycle (Fedele et al., 1989). The initial decrease of pain intensity in women with primary dysmenorrhea treated with placebo emphasizes the importance of assessing efficacy of treatment options over several cycles (for comparison, in the present case series Bryophyllum 50% tablets were usually taken for 3 months before reassessment).

Repurposing *B. pinnatum* for the treatment of dysmenorrhea presupposes that it is safe and well tolerated. With a view to safety, especially bufadienolides should be considered, since they are known for their cardiotoxic activity. However, cardiotoxicity of *B. pinnatum* and related plant species was only observed in animals that ingested a large amount of plants (McKenzie et al., 1987; Reppas, 1995). With a daily dose of 6 tablets, the intake of bufadienolides is very low (0.035 μg/kg of bersaldegenin-1,3,5-aceate) and below the amount shown to have toxic effects [see (Bachmann et al., 2017) and references therein]. In experiments using fresh myometrium and detrusor muscle, tissue viability was confirmed after the exposure to test compounds by assessing contractile activity after a wash out phase (Fürer et al., 2015b; Santos et al., 2018; Santos et al., 2019). Additionally, toxic effects were found in myometrial cell lines only when very high doses of press juice or fractions enriched with bufadienolides and flavonoids were used (Santos et al., 2018; Santos et al., 2019). In an *in vivo* study, the treatment of mice at supratherapeutic levels did not show any effect on liver enzymes, urea nitrogen, or alkaline phosphatase (Torres-Santos et al., 2003; Hosomi et al., 2022). Also the treatment of pregnant wistar rats with *B. pinnatum* showed no toxicity at therapeutic levels and merely reduced glucose levels (Hosomi et al., 2022). All clinical studies performed so far showed that treatment with *B. pinnatum* preparations can be considered as safe and well-tolerated, without severe adverse events and only occasional adverse events that included stomach-ache, diarrhea, and exanthema (Betschart et al., 2013; Simões-Wüst et al., 2018; Mirzayeva et al., 2023). Gastrointestinal side effects upon treatment with the best-investigated preparations of *B. pinnatum*, namely, Bryophyllum 50% tablets, are most probably related to the contained lactose in patients with lactose intolerance. This product is widely used in Switzerland, especially during pregnancy (more than 30% of pregnant women take Bryophyllum at some point during their pregnancy) (Fürer et al., 2015b; Gantner et al., 2021).

In a next step, the effectiveness of Bryophyllum 50% tablets for the treatment of dysmenorrhea should be investigated in a prospective clinical study with an appropriate patient number. While doing so, the following aspects should be taken into consideration: Firstly, the dosage of six tablets daily (chosen in our case series) seems to be suitable for treatment. Secondly, it is important to cover at least three cycles to avoid over-evaluation of placebo effects. Thirdly, and in view of the anti-inflammatory effects of *B. pinnatum* preparations, changes in inflammation-associated symptoms such as lower back pain, diarrhea, sleeping disorders and other psychological symptoms should be considered. Such symptoms are experienced by many women with dysmenorrhea and are likely to be associated with the high occurrence of prostaglandins in the body of menstruating women (Iacovides et al., 2015). Finally, it would be advisable to directly monitor quality of life. In a study conducted among university students in Spain, women with dysmenorrhea had a lower mean quality of life score than women who did not suffer from dysmenorrhea, even if no correlation between the pain score and the perceived quality of life was found (Fernández-Martínez et al., 2019).

The review of the literature described in the first part of the present work showed that the pharmacological and clinical effects of *B. pinnatum* preparations support its use in the treatment of dysmenorrhea. In the second part of this work, first cases of women tentatively treated with Bryophyllum 50% chewable tablets revealed symptom improvement and very good tolerability. Taken together, our work encourages further clinical investigation of the use of Bryophyllum 50% in the treatment of dysmenorrhea. As a next step, a prospective clinical study with higher patient number is under preparation.

## Data Availability

The original contributions presented in the study are included in the article, further inquiries can be directed to the corresponding author.

## Abbreviations

[Ca^2+^]_i_, Intracellular calcium concentration; AUC, Area under the curve; AVP, Arginine vasopressin; AVPR1a, Arginine vasopressin receptor 1a; COX-1/2, Cyclooxygenase 1/2; ERK1/2, Extracellular signal regulated kinase; hTERT-C3, Human telomerase reverse transcriptase cells; IL-1β, Interleukin 1β; JNK/SAPK, c-jun N-terminal kinase/stress-activated protein kinase; MAPK, Mitogen activated protein kinase; NF-κB-p65, Nuclear factor κ-light-chain-enhancer of activated B cells; NRS, Numeric rating scale; NSAID, Non steroidal antiinflammatory drugs; OT, Oxytocin; OTR, Oxytocin receptor; PG, Prostaglandin; PHM1-41 cells, Pregnant human myometrial cells 1-41; PRISMA-ScR, Preferred reporting items for systematic reviews and meta-analyses for scoping reviews; TNF-α, Tumor necrosis factor α; VEGF, Vascular endothelial growth factor.
